# Electronic strengthening mechanism of covalent Si via excess electron/hole doping

**DOI:** 10.1038/s41598-023-42676-z

**Published:** 2023-10-02

**Authors:** Hiroki Noda, Shumpei Sakaguchi, Ryoga Fujita, Susumu Minami, Hiroyuki Hirakata, Takahiro Shimada

**Affiliations:** Department of Mechanical Engineering and Science, Kyoto University, Kyoto-Daigaku Katsura, Nishikyo-Ku, Kyoto, 615-8540 Japan

**Keywords:** Mechanical properties, Electronic structure, Semiconductors

## Abstract

Brittle fracture of a covalent material is ultimately governed by the strength of the electronic bonds. Recently, attempts have been made to alter the mechanical properties including fracture strength by excess electron/hole doping. However, the underlying mechanics/mechanism of how these doped electrons/holes interact with the bond and changes its strength is yet to be revealed. Here, we perform first-principles density-functional theory calculations to clarify the effect of excess electrons/holes on the bonding strength of covalent Si. We demonstrate that the bond strength of Si decreases or increases monotonically in correspondence with the doping concentration. Surprisingly, change to the extent of 30–40% at the maximum feasible doping concentration could be observed. Furthermore, we demonstrated that the change in the covalent bond strength is determined by the bonding/antibonding state of the doped excess electrons/holes. In summary, this work explains the electronic strengthening mechanism of covalent Si from a quantum mechanical point of view and provides valuable insights into the electronic-level design of strength in covalent materials.

## Introduction

Silicon is the most well-known semiconductor that is used widely in various devices such as integrated circuits, liquid crystal displays, and solar cells^1–3^. On the one hand, the demand for semiconductor devices is continuing to rise worldwide in recent years owing to the growing demand to realize an advanced information society using Internet of Things (IoT) and Artificial Intelligence (AI) technologies. On the other hand, it is known that semiconductor materials, including Si, are susceptible to brittle fracture due to mechanical loading and strain caused by thermal expansion under adverse operating conditions, which is a major cause of fatal defects in devices^4–6^. Brittle materials often fail from defect heterostructures such as microcracks and voids, which act as fracture initiation points due to stress concentration, that grow and coalesce leading to total rupture of the material. For example, a singular stress field is formed near the crack tip, and crack failure occurs owing to unstable propagation of the crack accompanied by the breaking of interatomic bonds at the crack edge when a critical load is reached^7,8^. Notches and holes are also known as fracture initiation points. Microscopically, all these fracture phenomena are caused by the breaking or recombination of interatomic bonds in stress-concentrated areas, such as crack tips and notch bottoms. In fact, recent experimental and theoretical studies on the fracture of brittle materials such as silicon have shown that the initiation of unstable crack propagation and fracture at the bottom of a notch are both governed by a single atomic bond at the crack tip or notch root^9,10^. In other words, the macroscopic failure of materials is governed by atomic-level bond breaking and recombination, and the macroscopic material strength ultimately depends on the strength of the interatomic bonds. Considering this fact, by designing the strength of interatomic bonds, we could design the strength and fracture properties of macroscopic materials, contributing to reliable design of semiconductor devices.

Recently, it has been experimentally demonstrated that mechanical properties such as lattice constants, elastic constants, and strength can be changed by doping materials with excess electrons or holes^11–22^. For example, in a nanoindentation test using Si doped with B at a concentration of $1.0 \times 10^{21} \;{\text{cm}}^{ - 3}$^16^, the elastic modulus decreased by 7.5% in comparison to undoped Si. It was also reported that light irradiation induced excess electrons/holes, which changed the brittle/ductile properties of the fracture behavior of the material^17–19^. Oshima et al. demonstrated that ZnS exhibited brittle fracture under light irradiation while ductile fracture in a dark environment^17^. In addition, a spherical indenter indentation test showed that the bond strength of ZnO under shear loading was reduced by hole doping with electron-beam irradiation^20^. It has also been reported that Si and ZnS with excess electrons/holes induced by light or electron-beam irradiation show reduced or enhanced fracture toughness^21,22^. These results suggest that the electron-induced changes in the mechanical properties of the materials are due to changes in the mechanical properties of the bonds caused by charge doping. However, the effects and mechanisms of excess electrons and holes on the mechanical properties of bonds have not been studied in detail.

The ideal strength is defined as the maximum stress at the onset of unstable deformation when a defect-free perfect crystal is subjected to uniform loading, which has been the subject of much research because it is the most fundamental type of strength^23–27^. When a perfect crystal is subjected to uniform loading, all lattice sites deform uniformly and interatomic bonds are broken simultaneously, so that the ideal strength reflects the bond strength. In other words, by evaluating the ideal strength of a crystal with electron doping, the effect of excess electrons/holes on the strength of atomic bonds can be discussed.

In this study, we aimed to clarify the effect of excess electrons/holes on the bond strength by performing first-principles ideal tensile strength simulations on Si as a representative brittle covalent material. We show changes in the mechanical properties such as the lattice constant, elastic constants, and ideal tensile strength of Si due to excess electrons/holes doping. In addition, we discuss the electronic mechanism of why the bond strength changes dramatically by electron doping.

## Simulation models and procedure

First-principles density functional theory (DFT^28,29^) calculations were performed using the Vienna ab initio simulation package (VASP)^30,31^. The electronic wavefunction was expanded using a plane wave basis set. The cutoff energy of the plane wave was set to 400 eV for the tensile simulations. The effects of electrons in the nucleus and inner core were represented by the projector-augmented wave (PAW) method^32,33^, and the Si 3s^2^3p^2^ electrons were treated as the valence states. Well-converged $6\times 14\times 8$ Monkhorst–Pack^34^ *k*-point grids were used to evaluate the tensile strength. The Gaussian smearing method with widths of 0.1 eV and 0.03 eV was used for tensile and band structure calculations, and the tetrahedron smearing method with a width of 0.03 eV was used for COHP calculations. Various exchange–correlation functionals of LDA^35^, GGA-PBE^36^, GGA-PBEsol^37^, and HSE06^38,39^ were carefully tested. As shown in the Supplementary Materials, the preliminary calculations demonstrated that HSE06 and LDA well reproduced the experimental results, and were subsequently used in this study as well.

**Figure 1:**
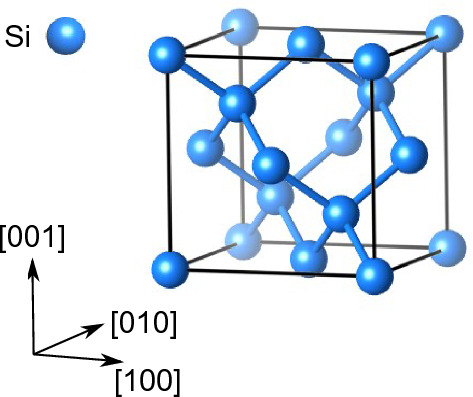
Crystal structure of Si with the diamond lattice.

Figure 1 shows the diamond structure of the Si crystal. Si is prone to brittle fracture on the (111) cleavage plane, and the covalent bonds between the (111) planes are perpendicular to the cleavage planes. To determine the strength of interatomic bonds, the bonds must be stretched along the bond direction. Therefore, uniaxial tensile loading in the [111] direction perpendicular to the cleavage (111) plane was applied to obtain the ideal tensile strength and the relevant strength of the covalent bond.

**Figure 2:**
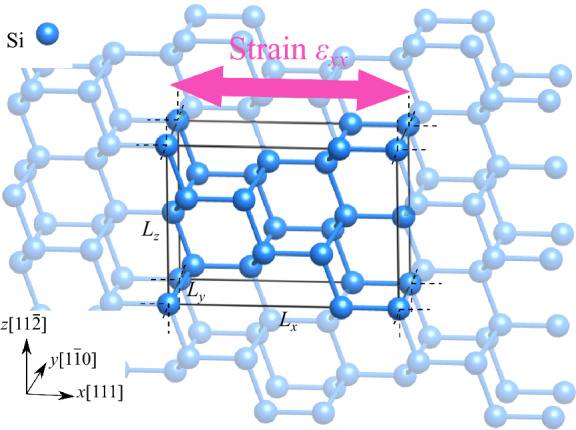
Loading condition of [111] tensile simulation of Si.

So far, Si single crystals with a maximum excess electron concentration *n*_*e*_ of $5.0\times {10}^{21}\,{\mathrm{cm}}^{-3}$ and maximum hole concentration ${p}_{h}$ of $5.0\times {10}^{21}\,{\mathrm{cm}}^{-3}$ have been experimentally fabricated^11^. In addition, experiments using an electric double layer^40–42^ have reported that it is possible to concentrate charge on the surface of ZnO^43^, which is a semiconductor like Si, at a high concentration of the same order of magnitude. With this in mind, we target Si with experimentally feasible excess electron or hole concentrations of 0 to $5.0\times {10}^{21}\,{\mathrm{cm}}^{-3}$. A model with excess electrons or holes was created by introducing or removing the number of electrons corresponding to the concentration in the cell. A homogeneous background charge was added to satisfy under conditions of electroneutrality^44^. It should be noted that these excess electron/hole doping are not doping additional atomic elements/impurities, but purely electron doping. In the case of dopants, because of the superposition of two effects, one due to the difference in ionic radii and the other electronic, there is a difficulty in discussing the individual effects in pure terms. In contrast, this study extracts only the electronic doping effect by purely doping electrons only, which allows a more detailed and separate discussion of the effects of dopants. Figure 2 shows a schematic of the tensile strain loading in the *x* direction for the [111] direction tensile loading analysis model. The following procedure was followed to calculate the ideal tensile strength (and strength of the Si bond). The cell vector corresponding to the [111] direction was elongated to provide a small tensile strain on the model. The other cell vectors and the atom positions were relaxed until the force acting on each atom was less than $1.0 \times 10^{ - 2}$ eV/Å and the stress component, other than the stress in the tensile direction, was less than 10 MPa. The tensile strain loading and structural relaxation were repeated so as to obtain the tensile stress–strain curves. The ideal tensile strength was obtained from the maximum tensile stress, and the critical tensile strain was obtained from the strain that reached the maximum tensile strength. Please refer to the Supplementary Material for additional details.

## Results

### Effect of excess electrons/holes on lattice parameters

**Figure 3:**
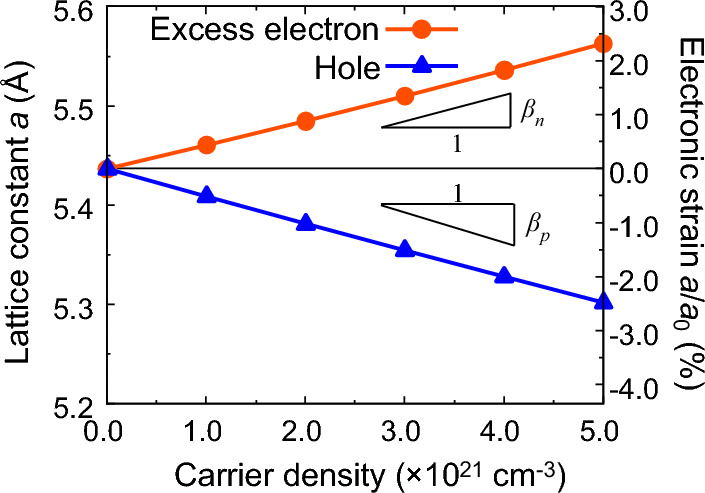
Lattice constant of doped Si as a function of carrier density of excess electron/hole.

Figure 3 shows the carrier concentration dependence of the Si lattice constant. Detailed data are presented in the Supplementary Material. Here, $a_{0}$ is the lattice constant of undoped Si. The lattice constant of excess electron doped Si is 5.460 Å at a concentration of $1.0 \times 10^{21} \;{\text{cm}}^{ - 3}$, which is 0.44% higher than that of undoped Si (5.437 Å). The increase in lattice constant via doping indicates that tensile electronic strain (strain induced by electron doping is generally called electronic strain^45^) is introduced by the excess electrons. As the tensile electronic strain is equivalent in the *x*-, *y*-, and *z*-directions, the bond length increases equivalently by maintaining the initial cubic diamond structure. Additionally, it can be observed that the lattice constant increased monotonically with the doping concentration, indicating that a larger tensile electronic strain was introduced by a higher electron concentration (Fig. 3). Conversely, in the hole doping case, the lattice constant decreases monotonically with increasing concentration thereby resulting in compressive electronic strain. These results indicate that the lattice constant monotonically increases with increasing excess electron concentration and decreases with increasing hole concentration resulting in either tensile or compressive electronic strain, respectively.

Moreover, the change in the lattice constant (electronic strain) has a nearly linear relationship with the doping concentration (Fig. 3). In a previous study, it was reported that the electronic strain $S_{ij}^{el} \left( {i,j = x,y,z} \right) = {\Delta }a/a_{0}$ and the doping concentration ∆*n* have a linear relationship as shown in the following equation^13^, which is in good agreement with the results of this study.

1 $$\begin{array}{*{20}c} {S_{ij}^{el} = \beta \Delta n\delta_{ij} ,} \\ \end{array}$$

where $\beta$ is a coefficient that expresses the relationship between the carrier density and electronic strain. To quantify this phenomenon, the coefficient of electron strain in relation to excess electron concentration was determined to be ${\beta }_{\mathrm{n}}=+4.36\times {10}^{-24}\,{\mathrm{cm}}^{3}$, and the coefficient for hole doping to be ${\beta }_{\mathrm{p}}=-5.13\times {10}^{-24}\,{\mathrm{cm}}^{3}$. Similarly, the pure effects of excess electrons/holes on the electronic strain of P-doped n-type Si and B-doped p-type Si were estimated in^14^ using experimentally obtained parameters while excluding the difference in the ionic radii of the dopants. The contribution of excess electrons in P-doped Si was evaluated to be ${\beta }_{\mathrm{n}}^{\mathrm{exp}}=+3.1\times {10}^{-24} \,{\mathrm{cm}}^{3}$, which was determined to be ${\beta }_{\mathrm{n}}=+4.36\times {10}^{-24} \,{\mathrm{cm}}^{3}$ in the present analysis, which is consistent with the experimental value in terms of both the positive value and order. In the same study, the contribution of holes in B-doped p-type Si was evaluated to be $\beta_{{\text{p}}}^{{{\text{exp}}}} = - 4.3 \times 10^{ - 24} {\text{cm}}^{3}$^14^. In the present analysis, ${\beta }_{\mathrm{p}}=-5.13\times {10}^{-24} \,{\mathrm{cm}}^{3}$, which is consistent with the experimental value in terms of both the negative value and the order. On the other hand, in the light-irradiated Si single crystal, the slope of the electronic strain with respect to the concentration of photoexcited excess electron/hole pairs, $\beta_{{{\text{pair}}}}^{{{\text{photo}}}} = - 9.5 \times 10^{ - 25} {\text{cm}}^{3}$^13^. Because the number of photoexcited excess electrons and holes are equal, ${\beta }_{\mathrm{pair}}$ is the sum of the excess electron contribution ${\beta }_{\mathrm{n}}$ and the hole contribution ${\beta }_{\mathrm{p}}$, and the following relationship holds good:

2 $$\begin{array}{*{20}c} {\beta_{{{\text{pair}}}} = \beta_{{\text{n}}} + \beta_{{\text{p}}} ,} \\ \end{array}$$

Using ${\beta }_{\mathrm{n}}$ and ${\beta }_{\mathrm{p}}$ in this work, we obtained the valued of ${\beta }_{\mathrm{pair}}=-7.7\times {10}^{-25} \,{\mathrm{cm}}^{3}$. This value is nearly equal to the experimental value of ${\beta }_{\mathrm{pair}}^{\mathrm{photo}}=-9.5\times {10}^{-25} \,\,{\mathrm{cm}}^{3}$. The comparison of ${\beta }_{\mathrm{n}}^{\mathrm{exp}}$, ${\beta }_{\mathrm{p}}^{\mathrm{exp}}$, and ${\beta }_{\mathrm{pair}}^{\mathrm{photo}}$ shows good agreement between the present analysis and previous data for the relationship between the excess electrons/holes doping concentration and electronic strain. Based on the aforesaid discussion, it can be concluded that this analysis accurately reproduces the effect of doped excess electrons/holes on the crystal structure of Si.

### Effect of excess electrons/holes on Young's Modulus

**Figure 4:**
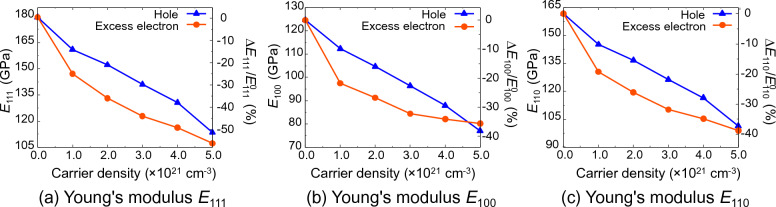
Young’s modulus of doped Si as a function of carrier density of excess electron/hole.* E*_111_, *E*_100_, *E*_110_ denote Young’s modulus in (**a**) [111], (**b**) [100], and (**c**) [110] directions, respectively.

Figure 4 shows the relationship between the Young's modulus in the [100], [110], and [111] directions and the doping concentration. Detailed data are shown in the Supplementary Material. Young's modulus ${E}_{111}$ is 147.0 GPa at excess electron concentration of $1.0\times {10}^{21} \,\,{\mathrm{cm}}^{-3}$, which is 18.1% lower than that of undoped Si (179.4 GPa). This trend is more pronounced at higher concentrations, and Young's modulus ${E}_{111}$ decreases with increasing excess electron concentration. Similar trends could be observed in the Young's moduli of ${E}_{100}$ and ${E}_{110}$ as well. In the case of hole doped Si also, in all directions the Young’s modulus decreases with increasing hole concentration, as is the case with excess electron doped Si. Therefore, the Young’s modulus of Si decreases with an increase in the concentration of excess electrons and holes. It was also experimentally reported that the elastic modulus decreased in the range of 4.39–10.49% from that of undoped Si using the indentation test for Si with B doping at a concentration of $1.0 \times 10^{21} {\text{cm}}^{ - 3}$^16^. This experimental measurement is in good agreement with our theoretical result of 9.9–10.3% decrease in Young's modulus of hole doped Si with the same concentration of $1.0\times {10}^{21} \,{\mathrm{cm}}^{-3}$. Hence, it can be concluded that this analysis accurately reproduces the effect of doped excess electrons/holes on the elastic properties of Si, and by controlling the electron doping concentration, the elastic properties of Si can be designed.

### Tensile strength and electronic structure of undoped Si

**Figure 5:**
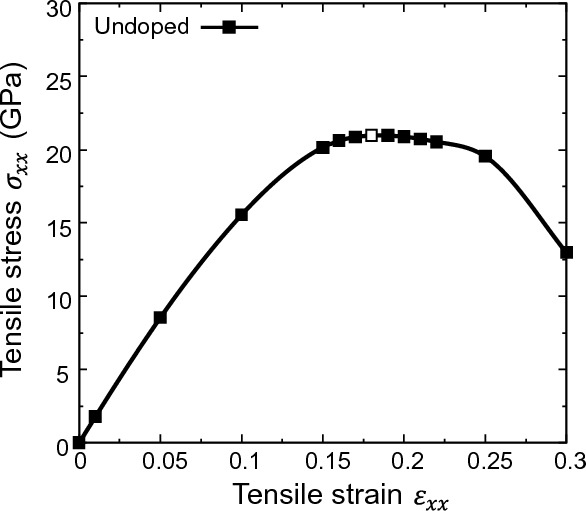
Tensile stress–strain curve of undoped Si. White-filled point indicates ideal tensile strength and critical tensile strain.

Figure 5 shows the stress–strain curve of the [111] tensile loading simulations for an undoped Si single crystal. The tensile stress increased monotonically with increasing tensile strain. The maximum stress ${\sigma }_{xx}$ = 20.98 GPa appears at a tensile strain of ${\varepsilon }_{xx}$ = 0.18. Thereafter, the tensile stress decreased as the tensile strain increased. Consequently, the ideal tensile strength ${\sigma }_{\mathrm{IS}}$ = 20.98 GPa, and the critical tensile strain ${\varepsilon }_{\mathrm{C}}$ = 0.18 were obtained and these results are in good agreement with those of previous reports^46,47^.

**Figure 6:**
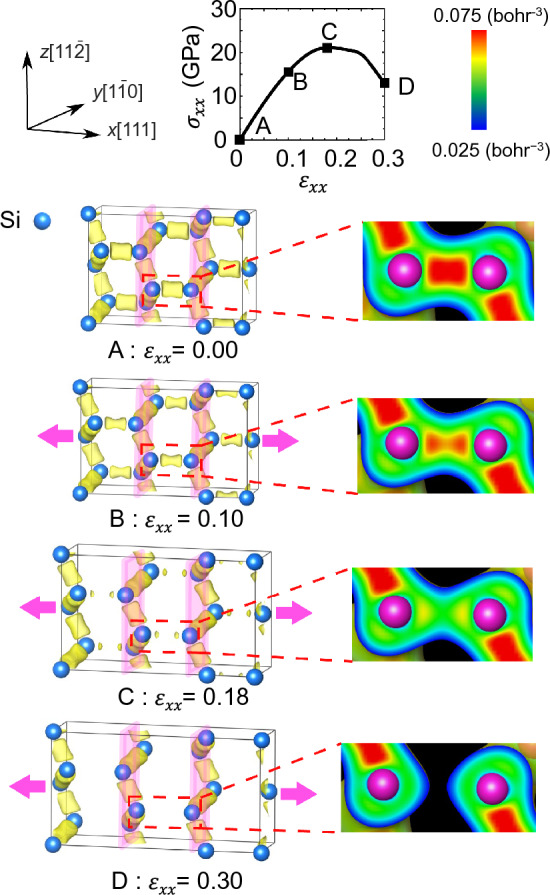
Charge density distribution of undoped Si during tensile simulation. The yellow surface represents the iso-surfaces of charge density of 0.06 ${\mathrm{bohr}}^{-3}$.

Figure 6 shows the charge density distribution in undoped Si under tensile loading in the [111] direction. At ${\varepsilon }_{xx}$ = 0.00, the electrons are densely distributed between adjacent Si atoms and form covalent bonds. At this stage, all bonds between these atoms can be confirmed to be equivalent. As the tensile strain increased, the charge density along the [111] covalent bond gradually decreased. After the tensile strain exceeded the critical strain of ${\varepsilon }_{\mathrm{C}}$ = 0.18, the charge density along the [111] bond got diluted and eventually the bonds were broken. During this deformation process, the bonds in the [111] direction are stretched and the electron density is diluted and the bonds are broken. On the other hand, the bonds in the [111] plane, which are perpendicular to the [111] direction, show almost no change in electron density when tensile strain is applied. In other words, the covalent bonds perpendicular to the loading direction (on the cleavage (111) plane) are maintained, whereas only those in the [111] direction are stretched and weakened. This indicates that the tensile stress as resistance to tensile loading is mainly carried by the [111] direction bonds. Therefore, the ideal tensile strength in the [111] direction obtained here can be considered to reflect the strength of the [111] direction covalent bond of Si.

### Effect of excess electrons/holes on tensile strength

**Figure 7:**
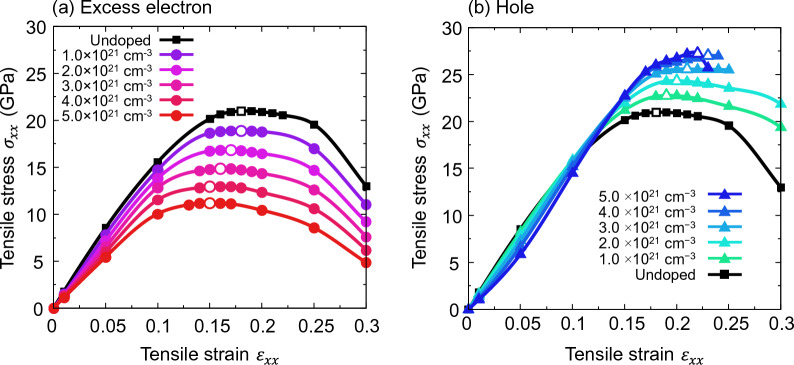
Tensile stress–strain curves of (**a**) excess electron and (**b**) hole doped Si. White-filled point indicates ideal tensile strength and critical tensile strain. It should be noted that these figures are similar to our previous work^22^, but with a different exchange–correlation term (LDA instead of PBEsol).

Figure 7 shows the stress–strain curve of excess electron/hole doped Si single crystals with a doping concentration of 1.0–5.0 × 10^21^ cm^−3^, along with the stress–strain curve for undoped Si, for comparison. Focusing on Si doped with an excess electron concentration of $1.0\times {10}^{21} \,{\mathrm{cm}}^{-3}$, the ideal tensile strength is ${\sigma }_{\mathrm{IS}}$ = 18.88 GPa. This value is lower than that of undoped Si (${\sigma }_{\mathrm{IS}}$ = 20.98 GPa) thus indicating that the ideal tensile strength of Si decreases with excess electron doping. At a higher concentration of $2.0\times {10}^{21} \,{\mathrm{cm}}^{-3}$, the ideal tensile strength is further reduced to ${\sigma }_{\mathrm{IS}}$ = 16.84 GPa, thus indicating that the ideal tensile strength decreases with increasing concentration of excess electrons. Comparing ${\sigma }_{\mathrm{IS}}$ for each excess electron concentration, it can be observed that the ideal tensile strength decreases monotonically as the excess electron concentration increases. For Si with a concentration of $5.0\times {10}^{21} \,{\mathrm{cm}}^{-3}$, the ideal tensile strength is ${\sigma }_{\mathrm{IS}}$ = 11.19 GPa, which is surprisingly 46.7% lower than that of the undoped Si, despite the feasible doping concentration.

On the other hand, focusing on Si doped with a hole concentration of $1.0\times {10}^{21} \,{\mathrm{cm}}^{-3}$, the ideal tensile strength is ${\sigma }_{\mathrm{IS}}$ = 22.78 GPa. This is higher than that of undoped Si (${\sigma }_{\mathrm{IS}}$ = 20.98 GPa), thus indicating that the ideal tensile strength of Si increases with hole doping. At a higher concentration of $2.0\times {10}^{21} \,{\mathrm{cm}}^{-3}$ , the ideal tensile strength increased further to ${\sigma }_{\mathrm{IS}}$ = 24.32 GPa, thus indicating that the ideal tensile strength increases with increasing concentration of holes. Comparing ${\sigma }_{\mathrm{IS}}$ for each hole concentration, the ideal tensile strength increases monotonically as the hole concentration increases. For Si with a hole concentration of $5.0\times {10}^{21} \,{\mathrm{cm}}^{-3}$, the ideal tensile strength is ${\sigma }_{\mathrm{IS}}$ = 27.23 GPa, which is surprisingly 29.8% higher than that of undoped Si, despite the feasible doping concentration. As described in “Tensile strength and electronic structure of undoped Si” section, the ideal tensile strength can be considered to correspond to the strength of the covalent Si bond. Therefore, the strength of the Si bond increases monotonically with hole concentration, whereas it decreases monotonically with excess electron concentration.

Given that many experiments have been reported with doping concentrations of the order of nearly $10^{21} \;{\text{cm}}^{ - 3}$, it is possible to reduce the strength of interatomic bonds in Si up to 46.7% through excess electron doping and increase it up to 29.8% through hole doping, using the experimentally feasible doping concentration. In fact, it has been reported experimentally that excess electrons and holes can have a significant effect on the strength properties of the material. In a previous study on the effect of holes on the ideal shear strength of ZnO, it was reported that holes experimentally reduced the shear strength by 26%, which is qualitatively consistent with the ideal shear strength change based on first-principles analysis^20^. Moreover, 31% decrease in fracture toughness was reported for ZnS with excess electrons/holes induced by photoirradiation^21^, and 4–11% increase in fracture toughness was reported for Si doped with holes by electron beam irradiation^22^. Because crack propagation in brittle materials is caused by the breaking of a single bond at the crack tip^48^ and the stress on the breaking of a bond at the crack tip corresponds to the ideal tensile strength^10^, the fracture toughness is considered to be related to the ideal tensile strength, which in turn corresponds to the bond strength. In fact, some studies have attempted to estimate fracture toughness from the stress–strain relationship of ideal strength calculations^49,50^. Based on the relationship between ideal tensile strength and fracture toughness, the increase in ideal tensile strength of hole doped Si is qualitatively consistent with the increase in fracture toughness of hole doped Si in the experiment. Therefore, the change in the ideal tensile strength due to electron doping of the material can be well evaluated by first-principles analysis.

**Figure 8:**
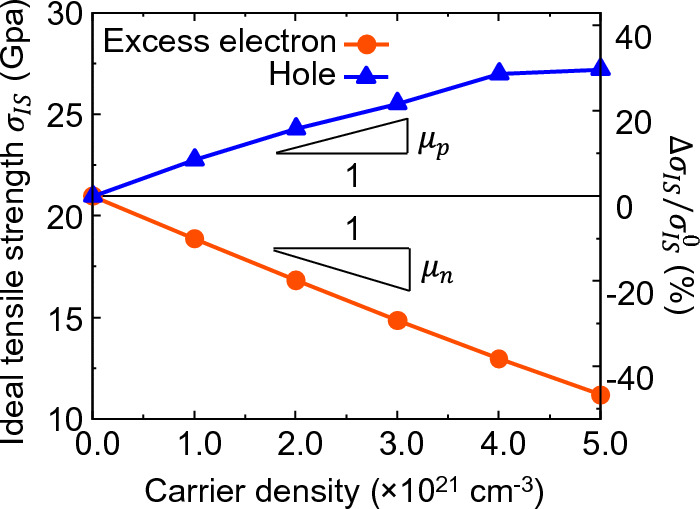
Ideal tensile strength of doped Si as a function of carrier density of excess electron/hole.

Figure 8 shows the relationship between the ideal tensile strength ${\sigma }_{\mathrm{IS}}$ and the doping concentration of excess electron- and hole-doped Si. Detailed data are shown in the Supplementary Material. Here, $\Delta \sigma_{{{\text{IS}}}}$ indicates the extent of change from the undoped Si. The ideal tensile strength decreases monotonically and displays a near-linear relationship with increasing excess electron concentration. Conversely, with increasing hole concentration, the ideal tensile strength increases monotonically and displays a near-linear relationship up to a hole concentration of $4.0 \times 10^{21} \;{\text{cm}}^{ - 3}$, whereas it exhibits a nonlinear increasing trend at higher concentrations. Based on the above results, the relationship between the ideal tensile strength and doping concentration can be approximated linearly as follows.

3 $$\begin{array}{*{20}c} {\Delta \sigma_{{{\text{IS}}}} /\sigma_{{{\text{IS}}}}^{0} = \mu \Delta n} \\ \end{array}$$

where $\sigma_{{{\text{IS}}}}^{0}$ is the ideal tensile strength without doping, $\Delta n$ is the excess electron or hole concentration (${\text{cm}}^{ - 3}$), and *µ* is a coefficient (${{\% }} \cdot {\text{cm}}^{3}$) that expresses the relationship between the excess electron or hole concentrations. When the coefficient for excess electron doped Si is $\mu_{{\text{n}}}$ and the coefficient for hole doped Si is $\mu_{{\text{p}}}$, we obtain $\mu_{{\text{n}}} = - 10.0 \times 10^{ - 21} {{\% }} \cdot {\text{cm}}^{3}$ and $\mu_{{\text{p}}} = + 8.6 \times 10^{ - 21} {{\% }} \cdot {\text{cm}}^{3}$ from the results of this analysis. In other words, the ideal tensile strength of Si can be decreased by excess electron doping and increased by hole doping, and the extent of change can be controlled by the excess electron or hole doping concentration. Here, the actual maximum level of doping corresponds to the degree to which the bond strength of Si can be controlled. Experimentally, doping levels of semiconductors on the order of ${10}^{21}\,{\mathrm{cm}}^{-3}$ have been achieved under heavily dope conditions^11,16,43,51^. Since the calculations in this study are performed with reference to this actual maximum experimental doping concentration, the Si bond strength can be designed from − 46.7 to + 29.8% by controlling the concentration of excess electrons or holes. Here, one of the most common causes of damage in semiconductors, including silicon, is thermal stress^5,6^. Repeated temperature changes during fabrication and operation induce stress, stemming from the differential thermal expansion coefficients between the semiconductor and either the substrate or other components. This stress can lead to the propagation of cracks from microscopic defects, notches, or bonding interfaces, ultimately resulting in fracture. As mentioned in the introduction, such fracture of brittle materials is governed by the breaking of a single bond^9,10^. For these vulnerabilities in semiconductor products, our strategies to improve bonding strength through doping might contribute to enhancing the product reliability. Furthermore, while this study focuses on the electronic effect, size effects also occur when introducing dopants. Related to lattice constants, the paper discussing the size and electronic effects of dopants has indicated that the size effect can contribute at a magnitude comparable to the electronic effect^14,52^. While elastic and strength properties do not directly relate to the lattice constants, the size effect might have an influence on par with the electronic effect reported in this paper. If this is the case, a variety of mechanical properties could be tailored by choosing suitable dopants that offer both size and electronic effects aligning with the desired mechanical properties.

It is noteworthy that in the case of hole doped Si, the Young’s modulus decreases while the ideal tensile strength increases, and the two mechanical properties display a contradictory response to hole doping. Adopting the classical theory of Frenkel^53^ and Orowan^54^ for approximating the stress–strain curve with a sinusoidal form, the ideal tensile and shear strengths can be expressed by the elastic properties (Young's modulus and transverse modulus) corresponding to each mode of deformation, as in the following equations. The critical strain is considered by referring to the study on ideal shear strength by Ogata et al.^55^.

$$\sigma_{{{\text{IS}}}} = \frac{{2E\varepsilon_{{\text{C}}} }}{\pi },\;\; \tau_{{{\text{IS}}}} = \frac{{2G\gamma_{{\text{C}}} }}{\pi }$$

where $E$ and $G$ are the Young's modulus and transverse modulus, respectively, and ${\varepsilon }_{\mathrm{C}}$ and ${\gamma }_{\mathrm{C}}$ are the critical tensile and shear strains, respectively. In the undoped case, substituting Young’s modulus and critical strain into the above equation yields ${\sigma }_{\mathrm{IS}}$ = 20.47 GPa, with an error of 2.0% from the calculated ideal tensile strength. Similarly, the error is in the range of 8.4–15.8% in the case of excess electron doping while it is in the range of 14.6–41.7% in the case of hole doping. The changes in the mechanical properties due to hole doping exhibit different characteristics from those expected from the Frenkel-Orowan theory. Conversely, Ogata et al. explained the reversal of the magnitude of the transverse modulus and ideal shear strength in copper and aluminum by focusing on the difference in deformation mechanisms^56^. From the above, it is suggested that the tensile deformation mechanism in the hole doped Si may show different characteristics from that in the undoped Si.

**Figure 9:**
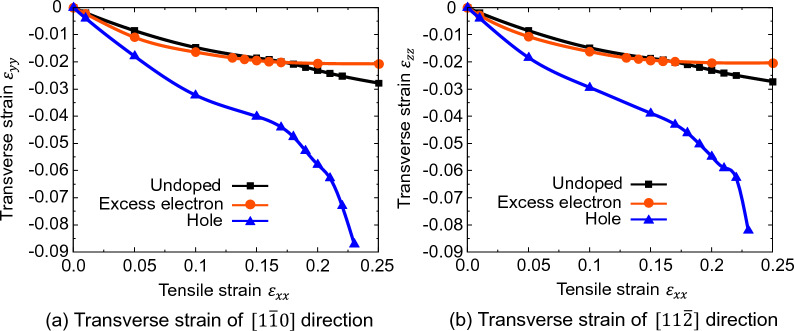
Transverse strain of Si on [111] tensile load. (**a**) Transverse strain ${\varepsilon }_{yy}$. (**b**) Transverse strain ${\varepsilon }_{zz}$.

Figure 9 shows the transverse strain ${\varepsilon }_{yy}$ and ${\varepsilon }_{zz}$ perpendicular to the tensile direction *x* [111] for Si doped with an excess electron and hole concentration of $5.0 \times {10}^{21}$ cm^3^. In undoped Si, compressive transverse strain occurs with applying tensile strain, indicating Poisson contraction. In excess electron doped Si, the transverse strain, or Poisson contraction, is the same as the undoped Si. On the other hand, in the hole doped Si, the transverse strain is 2 to 3 times higher in comparison to undoped Si, i.e., by applying tensile strain in [111] direction, hole doped Si undergoes larger compressive strain, larger Poisson contraction. This tendency is particularly pronounced in the high tensile strain region, and the tensile deformation of hole doped Si is different from that of undoped or excess electrons doped Si.

**Figure 10:**
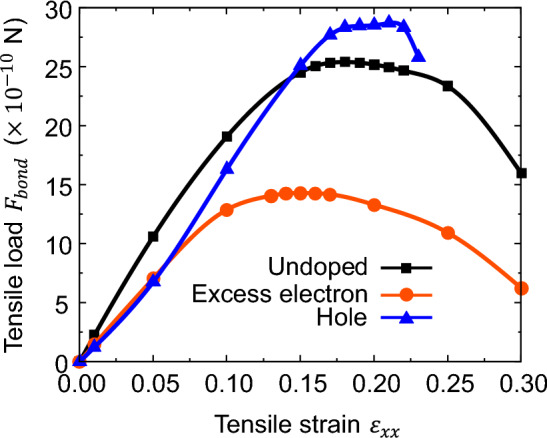
Tensile load of single [111] bond.

To discuss the effect of excess electron/hole on the strength of the bonds in Si, we evaluated the load per bond ${F}_{bond}$ by simply dividing the total load applied to the simulation cell by the number of Si–Si bonds along the [111] direction. Figure 10 shows the tensile load per bond ${F}_{bond}$ in the [111] direction. For undoped Si, the maximum tensile load per bond is $25.4\times {10}^{-10}\,\mathrm{ N}$. In contrast, the maximum tensile load for doped Si with an excess electron concentration of $5.0\times {10}^{21} \,{\mathrm{cm}}^{3}$ is $14.3\times {10}^{-10}\, \mathrm{N}$, which is approximately 43% lower than that for undoped Si. Conversely, the maximum tensile load for Si doped with a hole concentration of $5.0\times {10}^{21} \,{\mathrm{cm}}^{3}$ is $28.7\times {10}^{-10} \,\mathrm{N}$, which is approximately 13% higher than that for undoped Si. These results confirm that even when evaluating the strength per bond, the excess electrons decrease the strength and the holes increase the strength.

## Discussion

**Figure 11:**
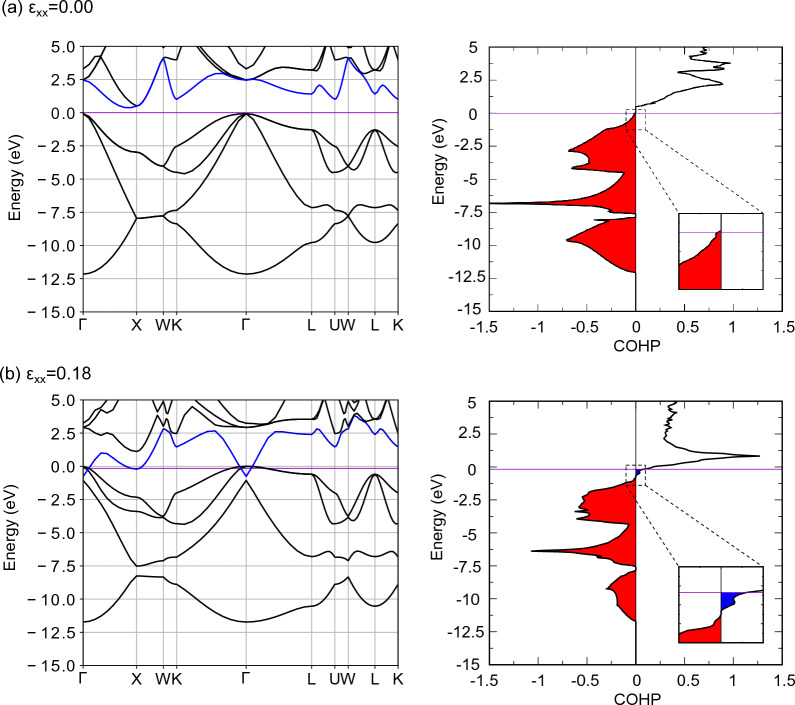
The band structure and COHP of undoped Si at several tensile strain. The Fermi level is represented by the purple line. The blue line in the band structure is the fifth band from the bottom band at zero strain. The red and blue areas in COHP indicate the bonding and antibonding states respectively.

Crystal Orbital Hamilton population (COHP) is a measure of the degree of chemical bonding in terms of energy, with more negative values for stable bonds with bonding orbitals and more positive values for unstable bonds with antibonding orbitals. COHP was analyzed using LOBSTER^57–60^ for those between Si atoms in the [111] direction, and Fig. 11 shows the band structure and COHP of undoped Si under tensile loading. In the unloaded state, Si electrons occupy all valence bands below the Fermi level (purple line in the figure). Because COHP is negative in the valence band (red region in the figure), it is a bonding orbital, and the electrons occupying this orbital form bonds that resist deformation. Because there are no orbitals in the band gap, electrons cannot occupy this energy band. The band in the conduction band is antibonding because COHP is positive, but in the ground state it is an empty band with no electrons, and no bonds are formed to promote deformation. When tensile loading is applied, the conduction band, indicated by the blue line in Fig. 11, shows a large decrease in energy with strain loading. It has been reported experimentally that the band gap of Si decreases with misfit strain loading^61^, which corresponds to the energy decrease in conduction band and band gap disappearance of Si in this calculation. As the energy of the antibonding orbital in the conduction band decreases, a positive blue region showing antibonding properties appears in the COHP below the Fermi level. In other words, the deformation promoting effect of the antibonding orbitals increases with tensile loading. Furthermore, the red region of COHP that is displaying the bonding properties, decreases with tensile loading. This implies that the bonds that resist the deformation caused by the bonding orbitals become weaker with tensile loading, and the deformation resistance effect of the bonding orbitals decreases. Therefore, the bond breaking of undoped Si is due to the weakening of the bonds due to the bonding orbitals (decrease in the deformation resistance effect) and the increase in the deformation promoting effect due to the antibonding orbitals.

**Figure 12:**
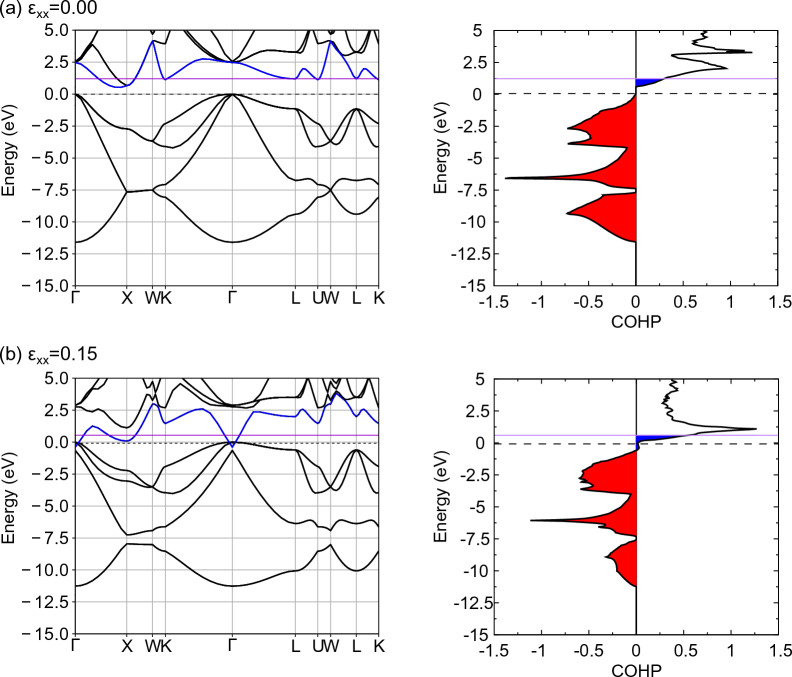
The band structure and COHP of excess electron doped Si at several tensile strains. The purple line indicates the Fermi level of excess electron doped Si. The dashed line indicates the Fermi level of undoped Si. The blue line in the band structure is the fifth band from the bottom band at zero strain. The red and blue areas in COHP indicate the bonding and antibonding states respectively.

**Figure 13:**
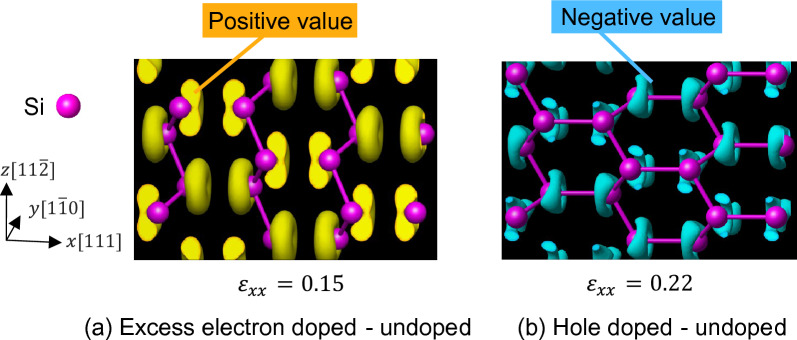
Difference of charge density between (**a**) excess electron doped and undoped (**b**) hole doped and undoped. Yellow and blue surfaces represent the iso-surfaces of difference of charge density of $1.0 \times 10^{ - 3} \;{\text{bohr}}^{ - 3}$ and $- 1.4 \times 10^{ - 3} \;{\text{bohr}}^{ - 3}$, respectively.

Figure 12 shows the band structure of excess electron doped Si under tensile loading and the COHP between Si atoms in the [111] direction. For comparison, the Fermi level of undoped Si is shown as a dashed line. In the unloaded state, the Fermi level crosses the conduction band, and a new blue region of COHP appears compared to the undoped Si. In other words, the excess electrons form antibonding orbitals, which have a deformation promoting effect compared to undoped Si, resulting in a decrease in Young's modulus. The band structure changes when tensile strain ${\varepsilon }_{xx}$ = 0.15 is applied. However, because electrons do not leave the bonding orbitals under tensile loading until the critical tensile strain is reached (red region in the COHP diagram), the bonds that bear the stress caused by the bonding orbitals are the same as those in undoped Si. Further, deformation is always promoted by the formation of antibonding orbitals by excess electrons (blue region in the COHP diagram). In other words, the decrease in bond strength of excess electron doped Si is caused by the deformation promoting effect of excess electrons occupying antibonding orbitals. Figure 13a shows the difference in electron density between excess electron doped Si and undoped Si under strain loading. The area surrounded by the yellow surface in the figure shows a positive value, indicating that the electron density is higher than that of undoped Si. Positive values appeared around the Si atoms, indicating that the electron density increased in this region. This distribution avoids the space between neighboring Si atoms. Thus, the excess electrons are not mainly involved in the bonding between Si atoms (bonding orbitals), which corresponds well with the band structure described above.

**Figure 14:**
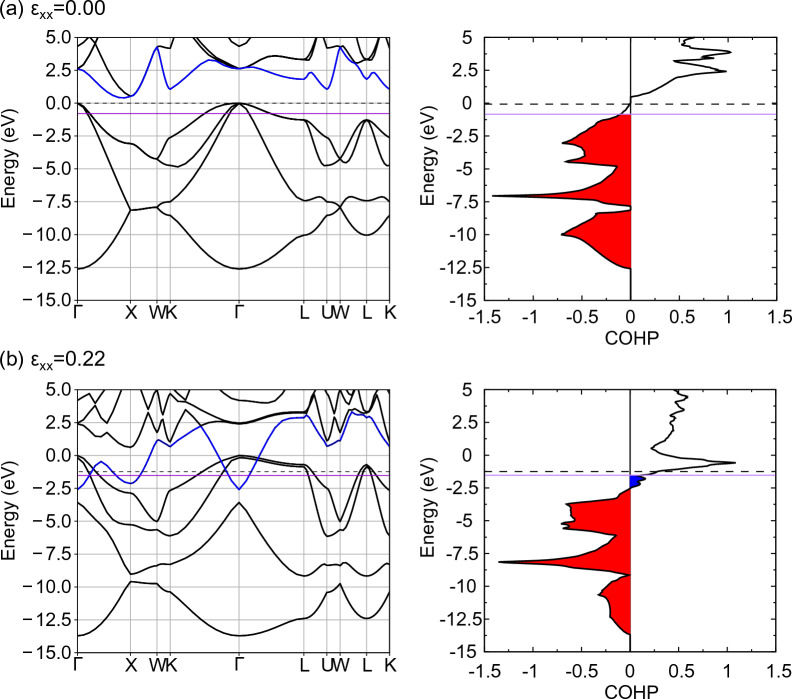
The band structure and COHP of hole doped Si at several tensile strains. The purple line indicates the Fermi level of hole doped Si. The dashed line indicates the Fermi level of undoped Si. The blue line in the band structure is the fifth band from the bottom band at zero strain. The red and blue areas in COHP indicate the bonding and antibonding states respectively.

Figure 14 shows the band structure of hole doped Si under tensile loading and the COHP between Si atoms in the [111] direction. For comparison, the Fermi level of undoped Si is shown as a dashed line. In the unloaded state, the Fermi level crosses the valence band, and the red region of the COHP is reduced compared with that of undoped Si. Holes occupy the bonding orbitals, i.e., electrons leave the bonding orbitals thereby resulting in weaker bonds between atoms than in the undoped Si, which leads to a corresponding decrease in Young's modulus. At a critical tensile strain of ${\varepsilon }_{xx}$ = 0.22 (Fig. 14b), holes occupy the antibonding orbitals, i.e., electrons leave the antibonding orbitals, and thus the deformation promoting effect decreases compared to the undoped Si. Given that the COHP of the bonding orbitals shown in the red region decreases with tensile loading, similar to the occurrence in undoped Si, the improvement in bond strength due to hole doping can be attributed to a reduction in the deformation promoting effect caused by holes occupying antibonding orbitals. In other words, the holes occupy different characteristic orbitals under no-loading and loading, resulting in a decrease in Young's modulus and improvement in bond strength that deviate from the Frenkel–Orowan theory. Figure 13b shows the difference in electron density between hole doped Si and undoped Si under strain loading. The area surrounded by the blue surface in the figure shows a negative value, indicating that the electron density is lower than that of undoped Si. Negative values appeared around the Si atoms, indicating that the electron density decreased in this region. This distribution avoids the space between neighboring Si atoms. In summary, it can be concluded that the holes are not primarily involved in the bonding between Si atoms (bonding orbitals), which corresponds well with the band structure described above.

## Conclusion

In summary, ideal tensile strength calculations using first-principles analysis to clarify the effect of excess electrons/holes on the bond strength of Si was performed to demonstrate that the bond strength of Si is decreased by excess electrons, whereas it is increased by holes. The strength decreases or increases monotonically with doping concentration, surprisingly showing a change of nearly 30–40% at the maximum feasible doping concentrations, which indicates that the strength can be controlled by the electronic conditions. Furthermore, through COHP analysis it has been demonstrated that the change in the bond strength of Si is determined by the bonding and antibonding state of the doped excess electrons and holes.

### Supplementary Information

Supplementary Information.

## Data Availability

The data that support the findings of this study are available from the corresponding author on reasonable request.
