# Supplementary material for: Electronic strengthening mechanism of covalent Si via excess electron/hole doping
Source: Sci Rep. 2023 Oct 2;13:16546. doi: 10.1038/s41598-023-42676-z (PMC10545711; doi:10.1038/s41598-023-42676-z)
Supplement: Supplementary file 1 — Supplementary Information. [file 41598_2023_42676_MOESM1_ESM.pdf]

Supplementary Materials

Electronic strengthening mechanism of covalent Si via excess electron/hole doping

Hiroki Noda[1,*], Shumpei Sakaguchi[1], Ryoga Fujita[1], Susumu Minami[1], Hiroyuki Hirakata[1], Takahiro Shimada[1,*]

1) Department of Mechanical Engineering and Science, Kyoto University, Kyoto-daigaku-Katsura, Nishikyo-ku, Kyoto 615-8540, Japan

*Corresponding author
E-mail: noda.hiroki.32a@kyoto-u.jp, shimada@me.kyoto-u.ac.jp

Selection of exchange-correlation terms in first-principles analysis for Si

Since the accuracy of the prediction of material properties by first-principles analysis is highly dependent on the evaluation of the exchange correlation term, the choice of the exchange correlation term approximation is important. In general, Local Density Approximation (LDA) [1] and Generalized Gradient Approximation (GGA) [2] are often used as exchange-correlation terms in first-principles analysis. Recently, PBEsol [3], which is an improved version of GGA proposed by Perdew, Burke, and Ernzerhof (PBE), has been proposed to accurately reproduce solid-state crystal structures. A hybrid functional method has also been proposed to accurately evaluate the band structure by incorporating the electron exchange of the Hartree-Fock (HF) method, which can correctly evaluate the exchange action, into the conventional exchange correlation terms such as LDA and GGA. In particular, HSE06 (Heyd-Scuseria-Ernzerhof) [4,5], one of these hybrid functional methods, has been reported to accurately evaluate the band gap of insulators and semiconductors [6]. Therefore, in this study, LDA, GGA-PBE, GGA-PBEsol, and HSE06 are candidates for the exchange-correlation term approximation.

Table S1 shows the lattice and elastic constants of undoped Si calculated using LDA, GGA-PBE, GGA-PBEsol, and HSE06 as exchange correlation terms. The experimental values of the lattice constant [7] and elastic constants [8] are also shown for comparison. where Δa and C_{ij} are the errors of the first-principles calculations of the lattice and elastic constants obtained using the respective exchange-correlation terms, relative to the experimental values. The calculated lattice constants have a maximum error of 0.69% with respect to the experimental values and are in good agreement with the experimental values regardless of the exchange correlation term used. Among them, GGA-PBEsol and HSE06 show particularly good agreement with the experimental values, with errors of 0.05% and 0.11%, respectively. For elastic constants, GGA-PBE has an error of 7.1 to 15.7 %, while LDA and GGA-PBEsol have errors of 4.2 to 5.6 % and 6.9 to 8.1 %, respectively, which are more accurate than GGA-PBE. HSE06 reproduces the elastic constants more accurately than the other exchange correlation terms, with an error of 0.6 to 6.6 % compared to the experimental values. The above results indicate that the crystal structure and mechanical properties (elastic properties) of Si can be evaluated accurately by selecting LDA, GGA-PBEsol, and HSE06 as the exchange correlation terms, and HSE06 reproduces them most accurately among them.

Table S1 Comparison of the theoretical values of lattice constant a and elastic constants C_{ij} of Si with the experimental values.

	LDA	GGA-PBE	GGA-PBEsol	HSE06	Experiment
a (Å)	5.402	5.469	5.434	5.437	5.431 [7]
Δa (%)	-0.54	0.69	0.05	0.11	-
C_{11} (GPa)	159.9	151.9	155.6	168.0	167 [8]
C_{12} (GPa)	62.2	54.8	60.3	60.7	65 [8]
C_{44} (GPa)	75.5	74.4	73.5	81.5	80 [8]
ΔC_{11} (%)	-4.3	-9.0	-6.9	0.6	-
ΔC_{12} (%)	-4.2	-15.7	-7.2	-6.6	-
ΔC_{44} (%)	-5.6	-7.1	-8.1	1.8	-

On the other hand, analysis using HSE06 requires a large amount of computation time compared to analysis using LDA or GGA-PBE. In the tensile load analysis and other analyses in this study, we need to perform analysis for multiple strain points for multiple Si doping concentrations. Therefore, it is difficult to perform detailed analyses that require a large number of data, such as stress-strain relationships, using HSE06 from the viewpoints of computational impossibility and time. As mentioned above, LDA and GGA-PBEsol correctly evaluate the crystal structure and mechanical properties of undoped Si. We will investigate whether these exchange-correlation terms can correctly reproduce the loading conditions during excess electron/hole doping.

Figure S1 shows the results of [111] tensile loading analysis for LDA and GGA-PBEsol. Figure S1(a), (b), (c), (d), and (e) show the results of [111] tensile loading analysis for undoped, excess electron concentration of 1.0×10^{21} cm^{-3}, 5.0×10^{21} cm^{-3}, and hole concentration of 1.0×10^{21} cm^{-3}, 5.0×10^{21} cm^{-3} , respectively. The stress-strain curves obtained by LDA and GGA-PBEsol are qualitatively and quantitatively almost identical to those of HSE06 in the tensile load analysis in the [111] direction for all doping concentrations. In other words, LDA and GGA-PBEsol can be used to accurately analyze the loading response of excess electron/hole doped Si. Therefore, in this study, LDA is used as the exchange correlation term in the elastic constants and [111] direction tensile loading analysis.

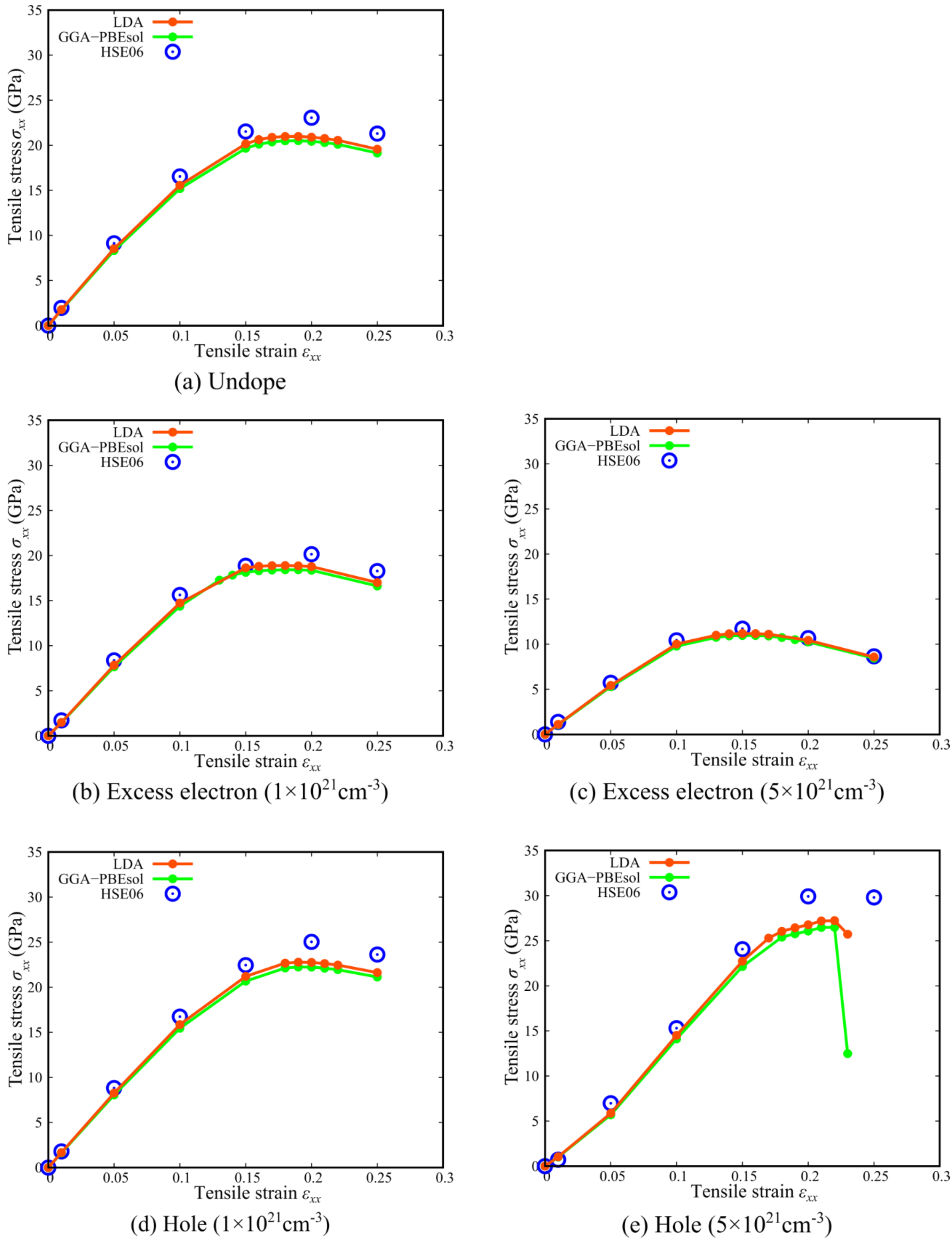

Figure S1 Tensile stress-strain curves of Si simulated with several exchange-correlation terms. (a) Undoped Si. (b) Excess electron doped Si at concentration of $1.0 \times 10^{21}\mathrm{cm}^{-3}$. (c) Excess electron doped Si at concentration of $5.0 \times 10^{21}\mathrm{cm}^{-3}$. (d) Hole doped Si at concentration of $1.0 \times 10^{21}\mathrm{cm}^{-3}$ (e) Hole doped Si at concentration of $5.0 \times 10^{21}\mathrm{cm}^{-3}$

Methodology

We performed first-principles density-functional theory (DFT [9,10]) calculations using the Vienna ab initio simulation package (VASP) [11,12]. The electronic wavefunction was expanded by a plane wave basis set. The cutoff energies of the plane wave were set to 600 eV for the evaluation of lattice and elastic constants and 400 eV for the tensile simulations. The effects of electrons in the nucleus and inner core were represented by the projector-augmented wave (PAW) method [13,14] and the Si $3s^2 3p^2$ electrons were treated as the valence states. We used well-converged $10 \times 10 \times 10$, $24 \times 24 \times 24$, $6 \times 14 \times 8$ Monkhorst-Pack [15] k-point grids for the evaluations of lattice constant, elastic constant, and tensile simulations, respectively. For the exchange correlation potential, HSE06 [4,5] was used for the evaluation of lattice constant and LDA [1] for the elastic constants and tensile simulations.

The model with excess electrons or holes was created by introducing or removing the number of electrons corresponding to the concentration in the cell. A homogeneous background charge is added to be performed under conditions of electroneutrality [16]. It should be noted that corrections are sometimes made when performing calculations on such charge doped materials. For example, in case charges are localized at defect sites, a correction to the energy is required due to errors caused by long-range electrostatic interactions [17]. On the other hand, in Si heavily doped with dopants such as B and P, infrared absorption is observed, which has been attributed to free carriers generated by the doping [18–20]. In other words, heavily doped charge in Si are generally delocalized and don't cause electrostatic interactions error. In this study, we deal with heavily doped charges such as excess electrons/holes that are delocalized in Si (i.e., charges are entirely distributed the system), so energetic correction, which are required for a localized defect system, is not necessary for our case. Therefore, in this paper, the effects of excess electrons/holes were analyzed without any energy correction.

Elastic constants and Young's modulus

The relationship between stress σ_{ij} and strain ε_{kl} of Si single crystal under linear elastic deformation is expressed by the following equation using the independent elastic constants C_{11}, C_{12}, and C_{44} in Si single crystal [21].

$$\begin{pmatrix} \sigma_{xx} \\ \sigma_{yy} \\ \sigma_{zz} \\ \tau_{yz} \\ \tau_{zx} \\ \tau_{xy} \end{pmatrix} = \begin{pmatrix} C_{11} & C_{12} & C_{12} & 0 & 0 & 0 \\ C_{12} & C_{11} & C_{12} & 0 & 0 & 0 \\ C_{12} & C_{12} & C_{11} & 0 & 0 & 0 \\ 0 & 0 & 0 & C_{44} & 0 & 0 \\ 0 & 0 & 0 & 0 & C_{44} & 0 \\ 0 & 0 & 0 & 0 & 0 & C_{44} \end{pmatrix} \begin{pmatrix} \varepsilon_{xx} \\ \varepsilon_{yy} \\ \varepsilon_{zz} \\ \gamma_{yz} \\ \gamma_{zx} \\ \gamma_{xy} \end{pmatrix}, \quad (1)$$

where ε_{ij} and γ_{ij} are the engineering strains and σ_{ij} and τ_{ij} are the corresponding stresses. As shown in Table S2, subscripts based on Voigt notation are used for elastic constants [22]

.

Table S2 Simplified subscripts using Voigt notation.

Face/Direction	xx	yy	zz	yz	zx	xy
Simplified subscripts	1	2	3	4	5	6

Now, when only tensile strain ε_{xx} is applied to this Si single crystal, the following stresses are obtained from equation (1).

$$\sigma_{xx} = C_{11}\varepsilon_{xx}, \tag{2}$$

$$\sigma_{yy} = C_{12}\, \varepsilon_{xx}, \tag{3}$$

$$(\sigma_{zz} = C_{12}\varepsilon_{xx}), \tag{4}$$

From equations (2) and (3), C_{11} and C_{12} can be obtained from the slopes of the stress-strain curves of the stresses σ_{xx} and σ_{yy} corresponding to a small tensile strain ε_{xx} loading, respectively. Similarly, when only shear strain γ_{xz} is applied to a Si single crystal, the following equation is obtained from equation (1).

$$\tau_{zx} = C_{44}\gamma_{zx}, \tag{5}$$

From equation (5), C_{44} can be obtained from the slope of the stress-strain curve of the stress τ_{xz} corresponding to a small shear strain γ_{xz} load. Therefore, to obtain the independent elastic constants C_{11}, C_{12}, and C_{44} for Si single crystals, it is sufficient to consider the tensile strain ε_{xx} and shear strain γ_{xz} loadings.

Now, Young's modulus of cubic single crystals has the following relationship with elastic constants C_{11}, C_{12}, and C_{44} [21].

$$\frac{1}{E_{100}} = \frac{C_{11} + C_{12}}{C_{11}^2 + C_{11}C_{12} - 2C_{12}^2}, \tag{6}$$

$$\frac{1}{E_{110}} = \frac{C_{11}}{2(C_{11}^2 + C_{11}C_{12} - 2C_{12}^2)} + \frac{1}{4C_{44}}, \tag{7}$$

$$\frac{1}{E_{111}} = \frac{C_{11} - C_{12}}{3(C_{11}^2 + C_{11}C_{12} - 2C_{12}^2)} + \frac{1}{3C_{44}}, \tag{8}$$

where E_{100}, E_{110}, and E_{111} are Young's moduli in the [100], [110], and [111] directions, respectively. Young's moduli E_{100}, E_{110}, and E_{111} of Si single crystal are evaluated by using these equations (6)~(8) and elastic constants C_{11}, C_{12}, and C_{44} obtained by the procedure described in the previous paragraphs.

Ideal tensile strength in [111] direction

Figure S2 shows a schematic of the tensile strain loading in the x direction for the [111] direction tensile loading analysis model. The conjugate gradient method was used to calculate the relaxation of the atomic structure until the force acting on each atom is less than 1.0×10^{-2} eV/Å and all stress components are less than 10 MPa to obtain the stable structure when no load is applied. Next, the cell vectors in the x direction are elongated to $(1 + \Delta\varepsilon_{xx})L_x$ to give a small tensile strain $\Delta\varepsilon_{xx}$ on the stable structure. The cell vectors L_y and L_z in the y and z directions and the internal atomic structure are relaxed so that the force acting on each atom is less than 1.0×10^{-2} eV/Å and the stress component in the tensile direction other than σ_{xx} is 10 MPa (σ_{ij} < 10 MPa; $ij \neq xx$). Then, the equilibrium state under tensile loading is obtained. The microstrain loading and structural relaxation are repeated until the tensile strain reaches $\varepsilon_{xx} = 0.40$, and a strain-controlled tensile loading analysis is performed to obtain the tensile stress-tensile strain relationship. The ideal tensile strength is obtained from the maximum tensile stress, and the critical tensile strain is obtained from the strain that reaches the maximum tensile strength.

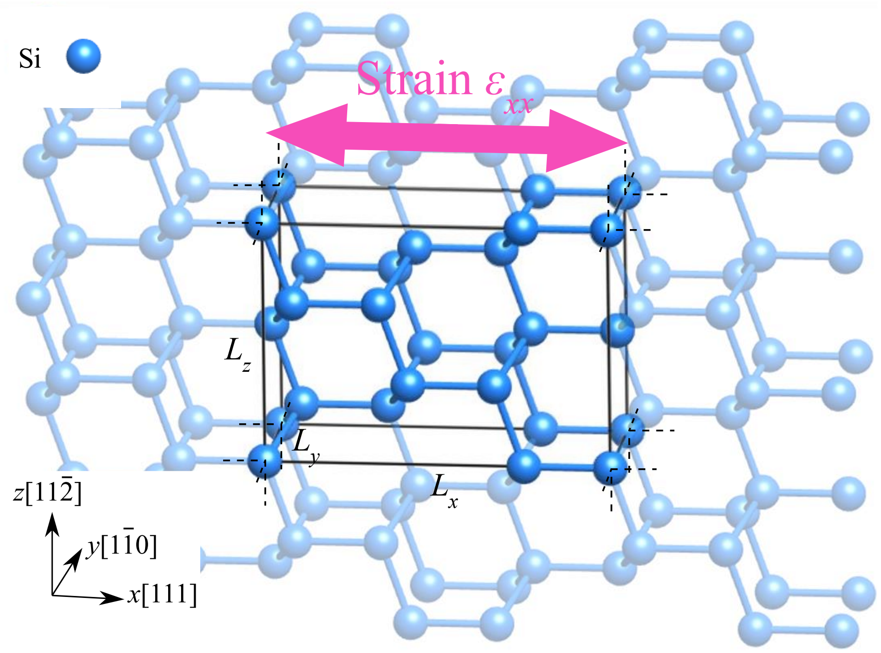

Figure S2 Loading condition of [111] tensile simulation of Si.

Table S3 Calculated lattice constant a of excess electron/hole doped Si.

	Undope	Excess Electron doping				
Concentration n_e (10^{21}cm^{-3})	0	1.0	2.0	3.0	4.0	5.0
Lattice constant a (Å)	5.437	5.460	5.485	5.510	5.536	5.562
$\Delta a/a_0$(%)	-	0.436	0.885	1.346	1.826	2.321
	Undope	Hole doping				
Concentration p_h (10^{21}cm^{-3})	0	1.0	2.0	3.0	4.0	5.0
Lattice constant a (Å)	5.437	5.409	5.381	5.355	5.328	5.302
$\Delta a/a_0$(%)	-	-0.513	-1.018	-1.513	-1.999	-2.479

Table S4 Calculated Young's modulus of excess electron/hole doped Si. E_{111}, E_{100}, E_{110} denote Young's modulus in [111], [100], and [110] directions, respectively.

	Undope	Excess Electron doping				
Concentration n_e (10^{21}cm^{-3})	0.0	1.0	2.0	3.0	4.0	5.0
E_{111} (GPa)	179.4	147.0	133.1	122.7	116.2	107.3
E_{100} (GPa)	124.6	97.5	91.3	84.5	82.1	80.2
E_{110} (GPa)	161.6	130.4	119.4	110.2	105.3	98.9
	Undope	Hole doping				
Concentration p_h (10^{21}cm^{-3})	0.0	1.0	2.0	3.0	4.0	5.0
E_{111} (GPa)	179.4	160.9	152.1	141.0	130.6	113.4
E_{100} (GPa)	124.6	112.3	104.7	96.4	87.9	77.0
E_{110} (GPa)	161.6	145.2	136.7	126.4	116.5	101.4

Table S5 Calculated ideal tensile strength of excess electron/hole doped Si.

	Undope	Excess Electron doping				
Concentration n_e ($10^{21}\mathrm{cm}^{-3}$)	0.0	1.0	2.0	3.0	4.0	5.0
Critical strain ε_C	0.18	0.18	0.17	0.16	0.16	0.15
Ideal strength σ_IS (GPa)	20.98	18.88	16.84	14.85	12.96	11.19
$\Delta\sigma_\mathrm{IS}/\sigma_\mathrm{IS}^0$ (%)	-	-10.0	-19.7	-29.2	-38.2	-46.7
	Undope	Hole doping				
Concentration p_h ($10^{21}\mathrm{cm}^{-3}$)	0.0	1.0	2.0	3.0	4.0	5.0
Critical strain ε_C	0.18	0.19	0.20	0.21	0.23	0.22
Ideal strength σ_IS (GPa)	20.98	22.78	24.32	25.55	27.03	27.23
$\Delta\sigma_\mathrm{IS}/\sigma_\mathrm{IS}^0$ (%)	-	8.6	15.9	21.8	28.8	29.8

References

[1] D. M. Ceperley and B. J. Alder, *Ground State of the Electron Gas by a Stochastic Method*, Phys. Rev. Lett. **45**, 566 (1980).

[2] J. P. Perdew, K. Burke, and M. Ernzerhof, *Generalized Gradient Approximation Made Simple*, Phys. Rev. Lett. **77**, 3865 (1996).

[3] J. P. Perdew, A. Ruzsinszky, G. I. Csonka, O. A. Vydrov, G. E. Scuseria, L. A. Constantin, X. Zhou, and K. Burke, *Restoring the Density-Gradient Expansion for Exchange in Solids and Surfaces*, Phys. Rev. Lett. **100**, 136406 (2008).

[4] J. Heyd, G. E. Scuseria, and M. Ernzerhof, *Hybrid Functionals Based on a Screened Coulomb Potential*, The Journal of Chemical Physics **118**, 8207 (2003).

[5] A. V. Krukau, O. A. Vydrov, A. F. Izmaylov, and G. E. Scuseria, *Influence of the Exchange Screening Parameter on the Performance of Screened Hybrid Functionals*, The Journal of Chemical Physics **125**, 224106 (2006).

[6] Y. Matsushita, K. Nakamura, and A. Oshiyama, *Comparative Study of Hybrid Functionals Applied to Structural and Electronic Properties of Semiconductors and Insulators*, Phys. Rev. B **84**, 075205 (2011).

[7] K. Nakayama, *Absolute Measurement of Lattice Constant*, Nihon Kessho Gakkaishi **17**, 303 (1975).

[8] H. J. McSkimin, *Measurement of Elastic Constants at Low Temperatures by Means of Ultrasonic Waves–Data for Silicon and Germanium Single Crystals, and for Fused Silica*, Journal of Applied Physics **24**, 988 (1953).

[9] P. Hohenberg and W. Kohn, *Inhomogeneous Electron Gas*, Phys. Rev. **136**, B864 (1964).

[10] W. Kohn and L. J. Sham, *Self-Consistent Equations Including Exchange and Correlation Effects*, Phys. Rev. **140**, A1133 (1965).

[11] G. Kresse and J. Hafner, *Ab Initio Molecular Dynamics for Liquid Metals*, Phys. Rev. B **47**, 558 (1993).

[12] G. Kresse and J. Furthmüller, *Efficient Iterative Schemes for* Ab Initio *Total-Energy Calculations Using a Plane-Wave Basis Set*, Phys. Rev. B **54**, 11169 (1996).

[13] P. E. Blöchl, *Projector Augmented-Wave Method*, Phys. Rev. B **50**, 17953 (1994).

[14] G. Kresse and D. Joubert, *From Ultrasoft Pseudopotentials to the Projector Augmented-Wave Method*, Phys. Rev. B **59**, 1758 (1999).

[15] H. J. Monkhorst and J. D. Pack, *Special Points for Brillouin-Zone Integrations*, Phys. Rev. B **13**, 5188 (1976).

[16] J. Neugebauer and M. Scheffler, *Adsorbate-Substrate and Adsorbate-Adsorbate Interactions of Na and K Adlayers on Al(111)*, Phys. Rev. B **46**, 16067 (1992).

[17] C. Freysoldt, J. Neugebauer, and C. G. Van De Walle, *Fully* Ab Initio *Finite-Size Corrections for Charged-Defect Supercell Calculations*, Phys. Rev. Lett. **102**, 016402 (2009).

[18] X. Chen, X. Pi, and D. Yang, *Critical Role of Dopant Location for P-Doped Si Nanocrystals*, J. Phys. Chem. C **115**, 661 (2011).

[19] A. Mimura, M. Fujii, S. Hayashi, D. Kovalev, and F. Koch, *Photoluminescence and Free-Electron Absorption in Heavily Phosphorus-Doped Si Nanocrystals*, Phys. Rev. B **62**, 12625 (2000).

[20] P. E. Schmid, *Optical Absorption in Heavily Doped Silicon*, Phys. Rev. B **23**, 5531 (1981).

[21] M. A. Hopcroft, W. D. Nix, and T. W. Kenny, *What Is the Young's Modulus of Silicon?*, J. Microelectromech. Syst. **19**, 229 (2010).

[22] M. Råsander and M. A. Moram, *Elastic Constants of the II–IV Nitride Semiconductors $MgSiN_2$, $MgGeN_2$ and $MgSnN_2$*, J. Phys. D: Appl. Phys. **51**, 375101 (2018).
